# Subcutaneous Fascial Bands—A Qualitative and Morphometric Analysis

**DOI:** 10.1371/journal.pone.0023987

**Published:** 2011-09-08

**Authors:** Weihui Li, Andrew C. Ahn

**Affiliations:** 1 Martinos Center for Biomedical Imaging, Massachusetts General Hospital, Charlestown, Massachusetts, United States of America; 2 Division of General Medicine and Primary Care, Beth Israel Deaconess Medical Center, Boston, Massachusetts, United States of America; University of Queensland, Australia

## Abstract

**Background:**

Although fascial bands within the subcutaneous (SQ) layer are commonly seen in ultrasound images, little is known about their functional role, much less their structural characteristics. This study's objective is to describe the morphological features of SQ fascial bands and to systematically evaluate the bands using image analyses tools and morphometric measures.

**Methods:**

In 28 healthy volunteers, ultrasound images were obtained at three body locations: the lateral aspect of the upper arm, medial aspect of the thigh and posterior aspect of lower leg. Using image analytical techniques, the total SQ band area, fascial band number, fascial band thickness, and SQ zone (layer) thickness were determined. In addition, the SQ spatial coherence was calculated based on the eigenvalues associated with the largest and smallest eigenvectors of the images.

**Results:**

Fascial bands at these sites were contiguous with the dermis and the epimysium forming an interconnected network within the subcutaneous tissue. Subcutaneous blood vessels were also frequently encased by these fascial bands. The total SQ fascial band area was greater at the thigh and calf compared to the arm and was unrelated to SQ layer (zone) thickness. The thigh was associated with highest average number of fascial bands while calf was associated with the greatest average fascial band thickness. Across body regions, greater SQ zone thickness was associated with thinner fascial bands. SQ coherence was significantly associated with SQ zone thickness and body location (calf with statistically greater coherence compared to arm).

**Conclusion:**

Fascial bands are structural bridges that mechanically link the skin, subcutaneous layer, and deeper muscle layers. This cohesive network also encases subcutaneous vessels and may indirectly mediate blood flow. The quantity and morphological characteristics of the SQ fascial band may reflect the composite mechanical forces experienced by the body part.

## Introduction

Subcutaneous (SQ) tissue is an adipose rich loose connective tissue layer lying underneath the dermal layer of the skin. It links the skin to the deeper muscular layers and bones [1]. It also acts as a storage depot for metabolic fuel and as a protective cushion for the deeper tissues and organs. For the past century, the main focus of the SQ region has been the adipocytes and traversing structures such as superficial nerves and vessels. The extracellular macromolecules such as collagenous and elastin fibers have garnered considerably less attention in the field of anatomy - partly due to the lack of clarity regarding their functional significance and partly due to difficulties in isolating them from the muscular fascia during dissections of an embalmed cadaver. With the increased availability and widespread use of ultrasonography, however, the existence of lateral band-like structures distinct from muscle, vessels or nerves is readily apparent within the subcutaneous layers. These bands appear as white, echogenic linear structures that span the width of the ultrasound image and have been matched to immune-histological staining of large collagenous fibers [2]. Anatomists and histologists have variably termed these extracellular fibers as “textus connectives compactus”, “Fascia superficialis”, “membrane layers”, and “Straffes Bindegewebe” [3]. For the purpose of clarity and uniformity, these collagenous bands will be termed “SQ fascial bands” in this manuscript.

Past studies have qualitatively assessed the characteristics of these SQ fascial bands and have described variability in both the number of bands and band thickness across body sites and across individuals [3]. To our knowledge, however, no study has quantitatively evaluated the morphological characteristics of these SQ fascial bands and identified factors associated with them. This study's objective is to systematically evaluate the morphological features of SQ fascial bands in healthy volunteers by analyzing ultrasound images obtained at three separate extremity sites – the upper arm, thigh, and calf. Image analytical techniques were used to quantify various aspects of SQ fascial bands - including the number of bands, band thickness, and total fascial band area – while also determining SQ zone thickness. We additionally evaluated the spatial anisotropy of the SQ zone with the use of a spatial coherence measure. The detailed description of the bands' morphology and their quantitative assessments may create the initial foundation for better understanding the physiological significance of these fascial bands.

## Methods

### Ethics Statement

This study was reviewed and approved by the institutional review board at the Beth Israel Deaconess Medical Center. Each study participant read and signed an informed consent form.

### Human Subject Recruitment

We recruited 28 subjects (19 females 9 males) in the study via flyers throughout Boston campus areas near Beth Israel Deaconess Medical Center and via postings in Craigslist (www.Craigslist.org). Subjects were excluded if they were under 18 years old or pregnant, used anticoagulation medications, had history of bleeding disorder, had an implanted ventricular defibrillator, had a chronic skin condition (such as eczema, psoriasis), or had a collagen disorder (scleroderma, mixed connective tissue disorder, Marfan's). Subjects' age was 28.6±7.9 (mean ± SD) years of BMI 23.5±3.6. Demographic representation was 23 non-Hispanic White, 1 Hispanic, and 4 Asian. Each subject was compensated for participation. The testing was performed in the General Clinical Research Center at the Beth Israel Deaconess Medical Center.

### Ultrasound Image Data Acquisition and Processing

**Figure 1:**
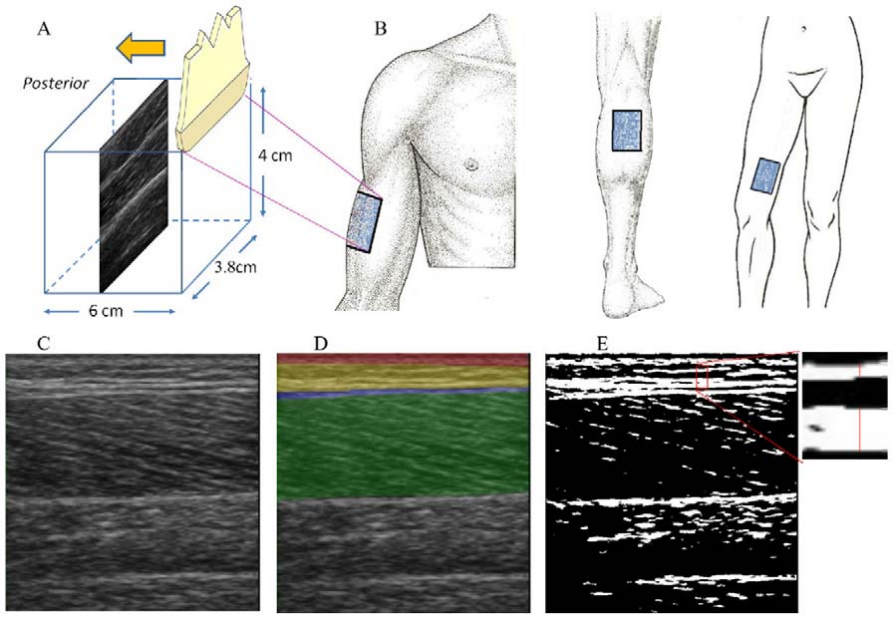
Three test segments with ultrasound image samples. (A) Illustration of ultrasound scanning longitudinal to the long axis of the extremities; (B) The locations where images were obtained: arm (left), calf (middle) and thigh (right); (C). Sample of ultrasound image; (D). Zones demarcated by color: dermal zone (red), SQ zone (yellow), epimysium zone (blue) and muscular zone (green); (E). Binary image with a pixel intensity threshold of 90.

A GE Logiq Book XP scanner (GE, Waukesha, Wisconsin) with a 38 mm linear array transducer (10MHz, 8L-RS) was used to image the subcutaneous region. Imaging depth was set at 40 mm and the focal depth was kept at 12.5 mm. Ultrasound transducer was placed in the direction longitudinal to the long axis of the extremities and swept anteriorly or posteriorly at a velocity of 1 cm/sec for a total of 6 seconds, as in Figure 1A. To standardize the measurements, transducer was held perpendicular to the skin. Minimal amounts of compression were applied on the skin to avoid deformation of the skin during ultrasound image acquisition. Recording mode was set to a cine loop setting – a 301 frame video loop was obtained for each sweep.

The videos were obtained at three different segments on the skin surface: the lateral aspect of the upper arm, medial aspect of the thigh and posterior aspect of lower leg, as previously reported [4] and shown in Figure 1B. These sites were predefined and localized according to adjacent body landmarks to reduce inter-subject measurement variability. Each video was then segmented to individual images (Figure 1C as an example) with Avidemux, a multi-platform video editor by Free Software Foundation Inc. (Boston, MA, USA). The segmented images were in jpeg format. Of the 301 images of each video, we evenly selected 6 images for imaging analysis. In cases where the imaging quality was in question (due to shadows or other imaging artifacts), the closest neighboring image without quality problems was chosen. We averaged the measurements from the 6 images and used this average for statistical analyses. In total, seventy five data sets from 450 images, 25 subjects, and 3 body sites (16 female and 9 male, 3 subjects' data were not analyzed due to poor image quality) were analyzed.

We used ImageJ software (National Institute of Health, USA) for imaging analysis. Images were first converted to 8-bit images with pixel intensity ranging from 0 to 255 (0 is no echogenicity and 255 is maximal echogenicity). The SQ layer was then marked out manually in the images to delineate the regions of interest (ROI), as shown in Figure 1D shaded with yellow. The red, blue and green zones in the figure were dermal, epimysial (dense connective tissue ensheathing the entire muscle) and muscular zones respectively, that were not included in the ROI. The depth and location of the SQ zone was confirmed by visualizing adjacent images within the video loop where the distinction between muscle and SQ zone was clear. Total pixel number in SQ was counted as the *total SQ zone area*. *SQ zone thickness* was calculated out by dividing total SQ zone area by the width of the SQ tissue.

We converted the images to binary images with a pixel intensity of 90 set as threshold. Pixels with intensity between 90 and 255 were regarded as echogenic. To eliminate the contribution of small, echogenic speckles to the total band area, high-intensity pixels with an aggregate area smaller than 20 pixels were categorized as non-echogenic. Figure 1E shows a sample of binary image. The threshold was determined by principal components analysis in JMP program (SAS institute Inc), as described below.

**Figure 2:**
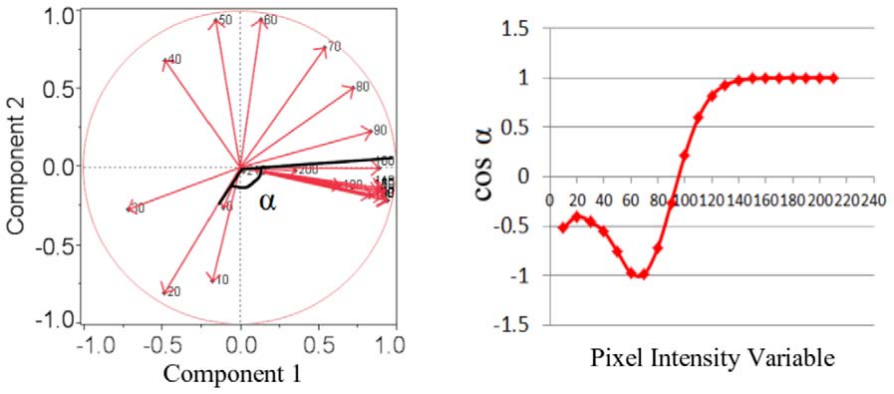
Determination of pixel intensity threshold by principal component analysis. (A). Eigenvectors of 22 decade variables plotted with respect to two principal components; (B). Value of cos(α) with respect to decade variables: α represents the angle formed between vector 1 (vector sum of all decade variables below designated decade variable) and vector 2 (vector sum of all decade variables above designated decade variable). (see text for detail).

In the binary image, the total number of echogenic pixels in SQ zone was referred to as *total fascial band area*. *Fascial band* was defined as a continuous, transverse line composed of high-intensity pixels. *Fascial band number* was the average number of fascial bands within the SQ zone and was determined by totaling the number of fascial band segments across all the pixel columns and dividing by the total number of columns. The inset of Figure 2F shows two sample fascial band segments, each marked with a red line. *Fascial band thickness* was estimated by dividing the total fascial band area by the fascial band number. *Fascial band density* was computed by dividing total fascial band area by total SQ zone area. In the image analyses, one-pixel was considered a unit. Each ultrasound image had dimensions of 390×412 pixels. With a linear array transducer width of 38 mm and imaging depth of 40 mm, we converted the pixel dimensions into metric units: 1 pixel  =  0.00956 mm^2^ in area and 1 pixel  =  0.097 mm in length.

We also calculated the coherence (Coh) for the SQ tissue with formula shown below: 
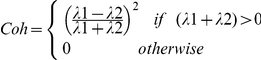
λ1 and λ2 are the first and second eigenvalues corresponding to the largest and smallest eigenvectors. Coherence is an indication of whether the local area is anisotropic. Coh is 1 when the local structure has one dominant orientation. The Coh is 0 when the local area is isotropic [5].

### Principal Components Analysis

Principal components analysis is a method that works with high-dimensional data and determines the vectors with greatest variances. By doing so, it reduces the dimensionality of the data and thus helps retrieve hidden patterns.

In this study, pixel intensities ranged from 0 to 221. No pixels in any of the ultrasound images had intensities above 221. The pixels were categorized into decades forming 22 variables. Each decade variable contained the number of pixels whose intensities fit within a specific range (e.g. 0–9 for the first decade variable) for each ultrasound image. These variables were used for the principal components analysis.

Our aim was to objectively categorize the images into high echogenicity (fascial band) and low echogenicity areas (non-band tissue). This implied the existence of two principal components. Figure 2A displays the loading plot from JMP and shows the eigenvector coordinates of each decade variable on the two-principal-components axes. To identify the most appropriate intensity threshold for differentiating echogenic and non-echogenic tissue, each decade variable was systematically used to divide all the variables into two groups. A summed vector was computed for each group, represented by the two thick black lines as shown in Figure 2A as an example (in this case, the summed vectors represent the first four vectors in one group and the remaining 18 vectors in the other group). These two vectors formed an angel α. Figure 2B showed the cosine values of the angle α for each decade variable, and the decade variable associated with the smallest magnitude of cos(α) was considered the optimal intensity threshold (α nearing 90 degrees indicates independence between the two vectors). In Figure 2B, cos(α) crosses the x-axis between pixel intensity variable 90 and 100. For this reason, pixel intensity threshold of 90 was chosen: pixels with intensity below 90 were treated non-echogenic and pixels with intensity above 90 were considered echogenic tissue.

### Statistical analysis

The study includes repeated measurements at three different locations on each subject for total 28 volunteers. With location as the dependent variable within each subject, different variables were analyzed to explore their location dependence with least-square means method. Pairwise comparison was also performed and adjusted with SCHEFFE algorithm [6]. To demonstrate the relationships between SQ zone variables, we conducted a bivariate linear fit. We used multilevel mixed model in determining the effect of location, SQ collagen band thickness, SQ collagen band number and SQ zone thickness on SQ coherence. The p value of 0.05 was set as the significant level.

## Results

### Structural morphologies of SQ collagen band

**Figure 3:**
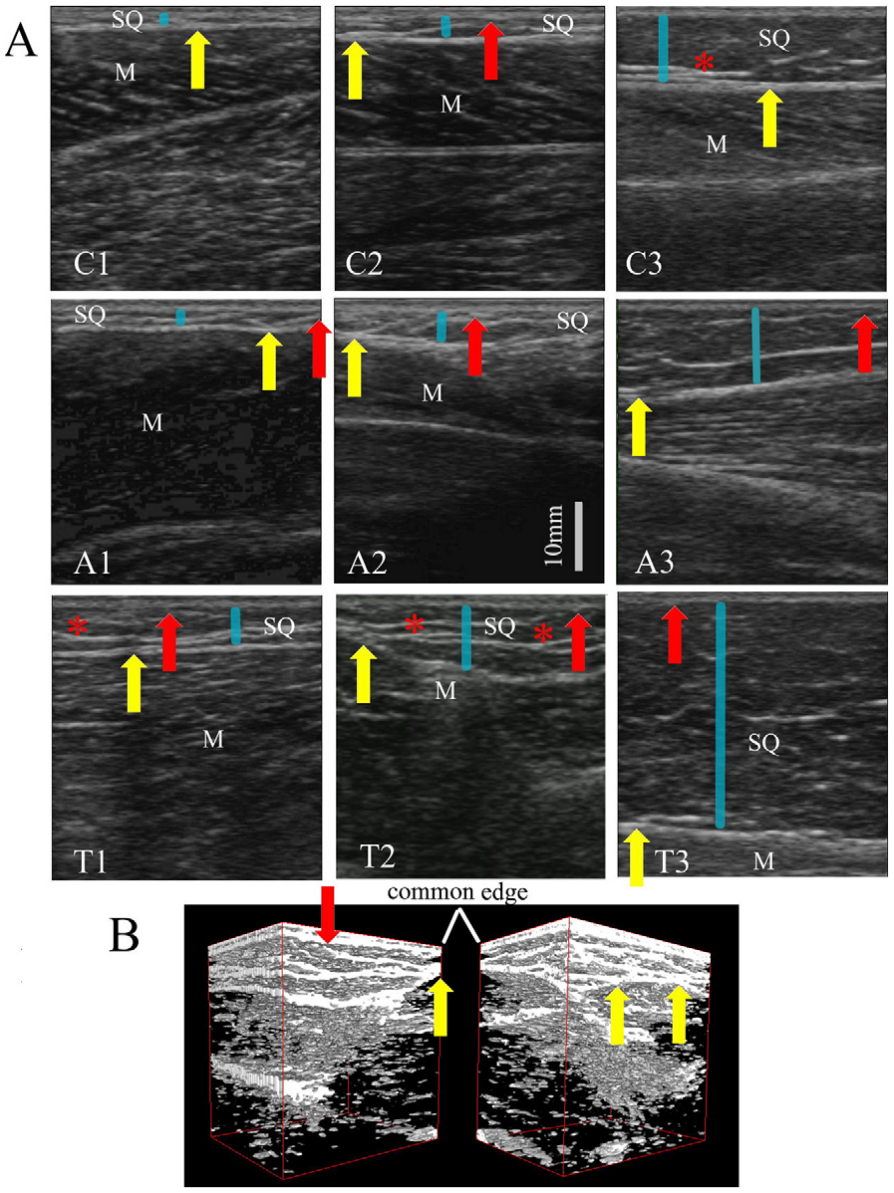
Ultrasound images showing the morphological characteristics of fascial bands. (A). Ultrasound images from calf, arm and thigh; (B). Three dimensional reconstruction of a video sweep, visualized from two different perspective angles.

Figure 3A shows sample ultrasound images from calf (panel C1, C2, C3), arm (panel A1, A2, A3) and thigh (panel T1, T2, T3) from different subjects. “M” and “SQ” in the figure labels the muscular layer and SQ layer respectively. Vertical blue lines are visual representation of the average SQ zone thickness. The layer above the top of the blue line is the dermal layer; the echogenic layer directly below the SQ zone is the epimysium layer.

SQ zone thickness (the length of the blue lines) greatly varied across individuals. For example, in the calf images, the SQ zone in C1 was approximately 2 mm while it was approximately 10 mm in panel C3. Similarly, in the arm and thigh images, SQ zone in panel A1 (about 3 mm) and T1 (about 5 mm) were thinner when compared to SQ zones in A3 (about 10 mm) and T3 (about 35 mm).

The morphology of fascial bands also differed across subjects. Fascial bands in panel A1 and T1 were generally linear and coherent whereas the bands in panel T3 were fragmented and curved. In general, individuals with decreased SQ zone thickness had more linear and less fragmented fascial bands compared to individuals with thicker SQ zones. The fascial band number also varied across individuals. More than two distinct echogenic bands are seen in panel T2 and only one relatively dimmer and less contiguous band is present in panel A1. In many cases, the SQ fascial bands are found aggregated close to the epimysium and less frequently near the dermis (C3, T1, T3 as examples). Among these images, some fascial bands are branched (below the red stars) while others are not.

The SQ fascial bands are, in many cases, contiguous with the epimysium and dermis, as demonstrated in Figure 3A. Red arrows indicate the site where SQ fascia plane extends towards the dermis, and the yellow arrows point to sites where epimysium and SQ fascial bands are fused. Figure 3B shows a three-dimensional reconstruction of serial binary images obtained from a video sweep and visualized from two different perspective angles. Again, the yellow and red arrows point to sites where the SQ fascial bands attach to the epimysium and dermis, respectively.

**Figure 4:**
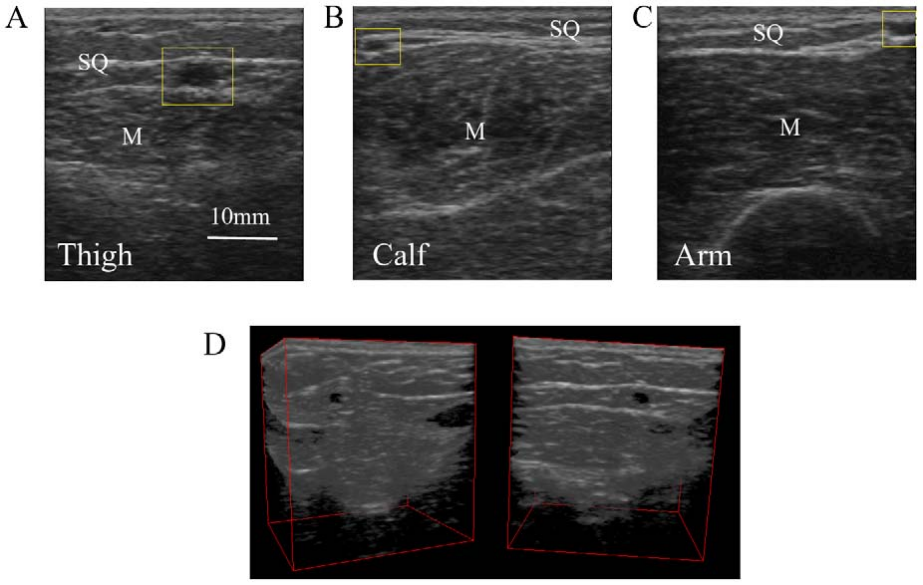
Ultrasound images showing the subcutaneous blood vessels encapsulated by fascial bands. (A). From the site of thigh; (B). From the site of calf ; (C) From the site of arm; (D). Three-dimensional reconstruction of a site with a vessel.

Multiple images revealed that subcutaneous veins are often encased by the fascial bands – either between SQ fascial bands or between the SQ fascial bands and epimysium. Figure 4A, 4B, 4C show sample images from the thigh, calf and arm. Figure 4D shows a cubic tissue reconstructed from the video sweep associated with Figure 4A. These two panels were viewed from two opposite direction with vein revealed as a black tunnel (also see “Video S1”). Within a span of only 6 cm, the fascial band's structural relationship with the vein can vary. As seen in the right panel of Figure 4D, the vein is located between two parallel, coherent fascial bands while, in the left panel, the fascial bands appears not to be as cohesive.

### Quantitative analysis of SQ fascial bands

**Figure 5:**
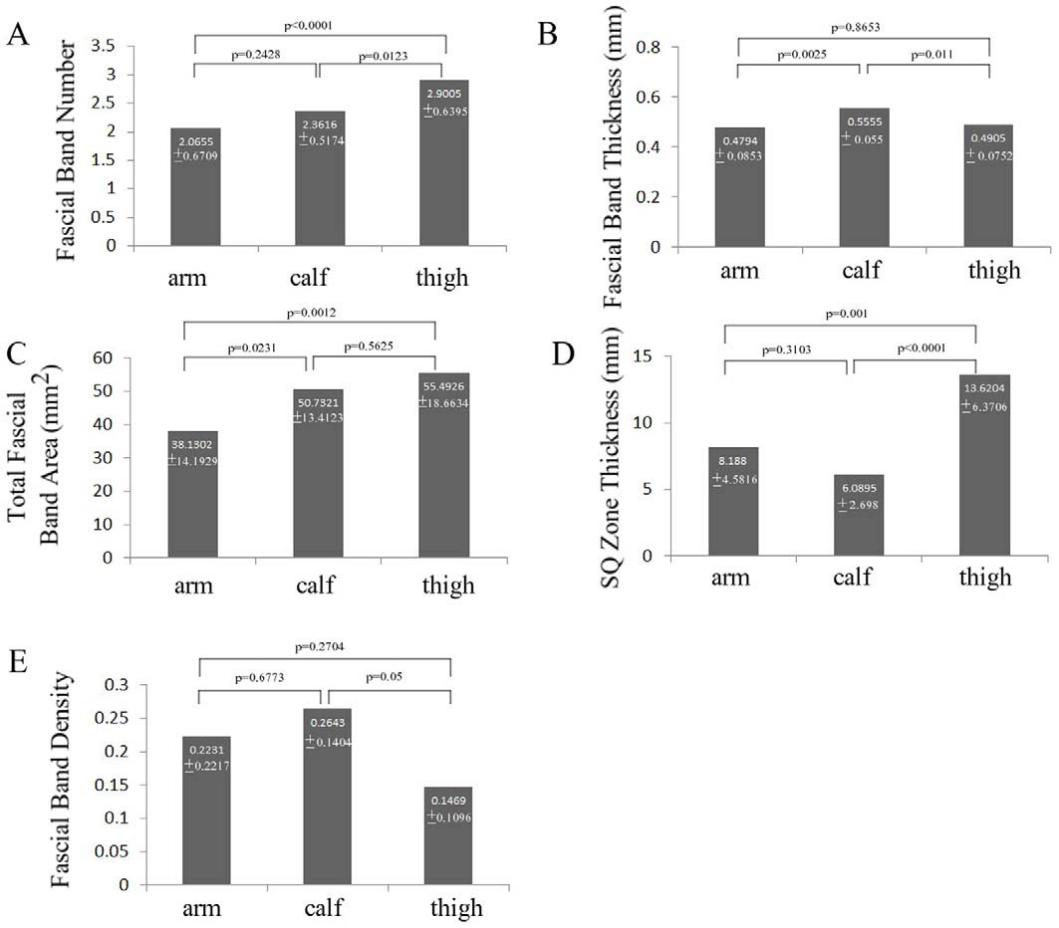
Body-location dependence of SQ zone variables with pairwise comparisons.

Table 1 lists the mean and standard deviations for the variables of fascial band number, fascial band thickness, total fascial band area, SQ zone thickness, and fascial band density regardless of locations. Their dependence on body site is presented in Figure 5 with pairwise comparison p values.

The thigh was associated with statistically greater number of SQ fascial bands when compared to the calf and arm (Figure 5A); SQ fascial band thickness was significantly greater at the calf compared to that of the arm and thigh (Figure 5B); the thigh had the greatest amount of total fascial band area while the arm had the least (Figure 5C).

**Table 1:** Means and standard deviations of ultrasound-image derived variables.

Variables	Mean	Std Dev
Fascial Band Number	2.44	0.7
Fascial Band Thickness (mm)	0.51	0.08
Total Fascial Band Area (mm^2^)	48.12	17.06
SQ Zone Thickness (mm)	9.3	5.7
Fascial Band Density	0.21	0.17

Figure 5D shows the mean thickness of the SQ zone at different body sites. SQ zone was thickest at the thigh and thinnest at the calf. The thigh was associated with a statistically greater SQ zone thickness compared to the arm and calf. Body site dependence of fascial band density is presented in Figure 5E. Only the pairwise comparison between the calf and thigh was statistically significant.

**Figure 6:**
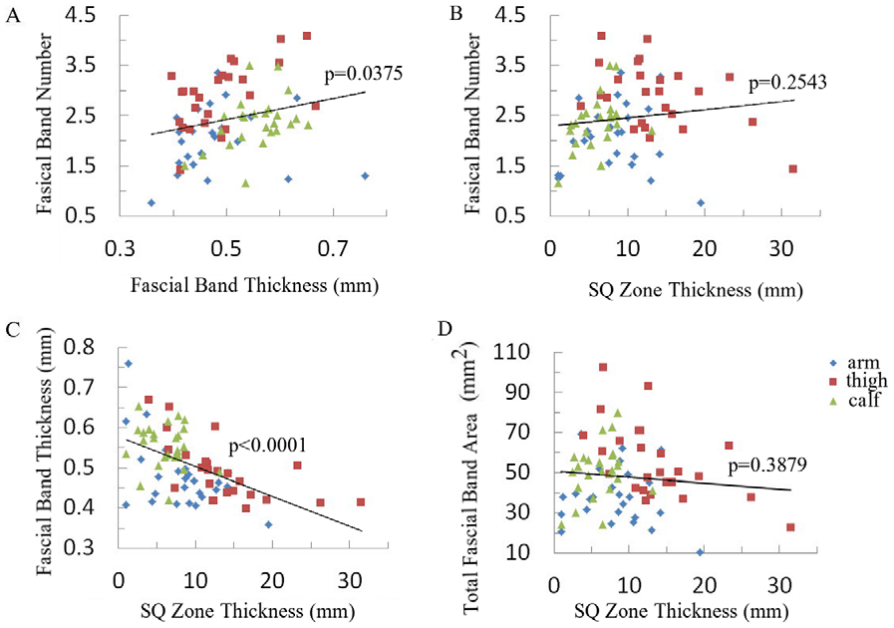
Bivariate linear fit between SQ zone variables.

Figure 6 shows the bivariate linear fit between the various fascial band variables in the SQ zone. Data from arm, thigh and calf were color coded as shown in the figure. There was a significant direct relationship between fascial band thickness and fascial band number (p = 0.0375). Fascial band number was not significantly associated with SQ zone thickness (p = 0.2543). There was significant correlations between fascial band thickness and SQ zone thickness (p<0.0001). The association between total total fascial band area and SQ zone thickness was also not statistically significant (p = 0.3879).

**Figure 7:**
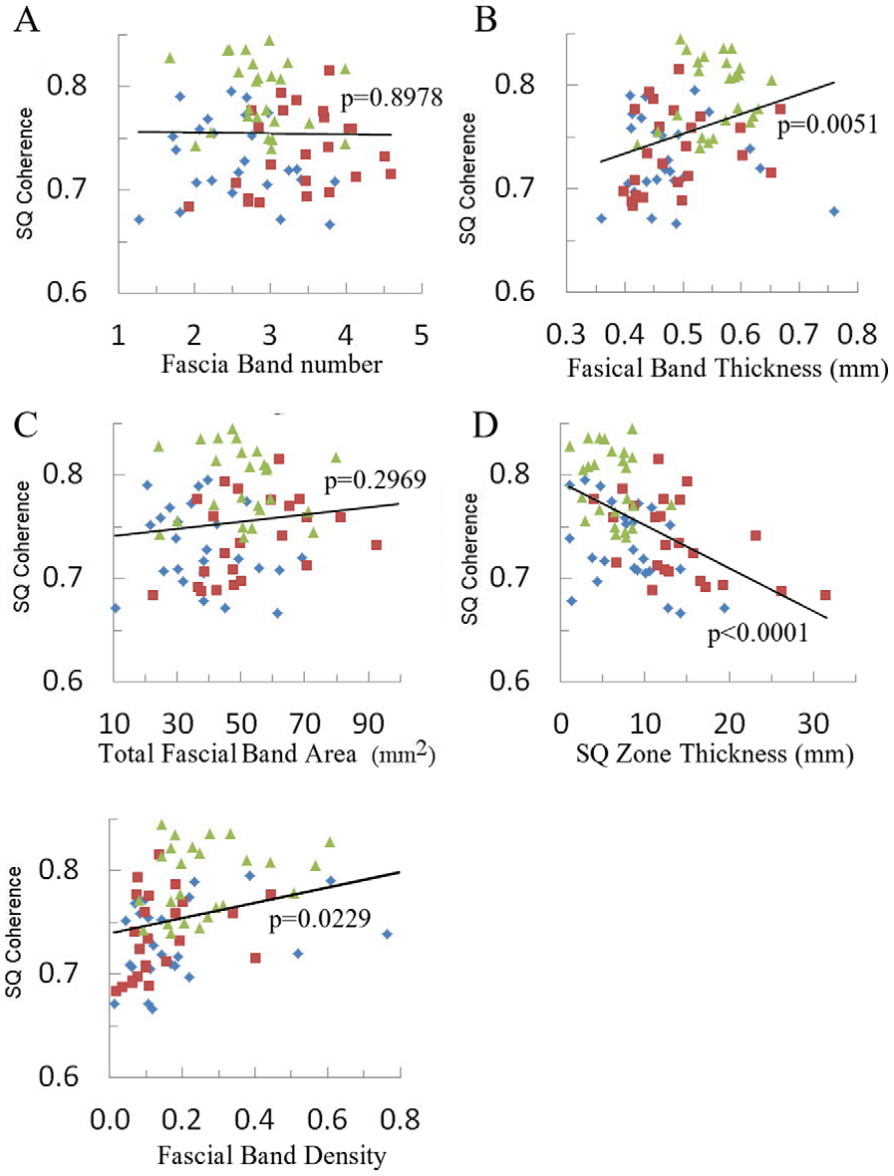
Bivariate linear fit between SQ coherence and various SQ zone variables. Green represents the data from arm, red from thigh and blue from calf.

The bivariate relationship between SQ coherence and various SQ variables - including fascial band number, fascial band thickness, total fascial band area, SQ zone thickness and fascial band density are presented in Figure 7. SQ coherence was not significantly associated with fascial band number (p = 0.8978) or total fascial band area (p = 0.2969), but was positively associated with fascial band thickness (p = 0.0051), fascial band density (p = 0.0229) and negatively correlated with SQ zone thickness (p<0.0001).

When SQ coherence was modeled in a multilevel mixed model with location, fascial band number, fascial band thickness and SQ zone thickness as independent variables, body site (p<0.0001) and SQ zone thickness (p<0.0001) were statistically significant factors (Table 2) while fascial band number and fascial band thickness were not (p = 0.6906 and 0.225 respectively). SQ coherence was greatest on the calf and least on the arm (Table 3). SQ coherence on the thigh was 0.711, significantly greater than on the arm (p = 0.0155) and comparatively reduced than on the calf (p = 0.3116).

**Table 2:** Factors associated with SQ coherence: results from a multilevel model with SQ coherence as dependent variable.

Variables	Intercept	Slope	p value
Locaiton			<0.0001
arm	0.76501		
calf	0.82475		
thigh	0.8045		
Fascial Band Number		-0.00291	0.6906
Fascial Band Thickness (mm)		-0.08691	0.225
SQ Zone Thickness (mm)		-0.00453	<0.0001

**Table 3:** Pair-wise comparisons of SQ coherence between body locations after accounting for other image derived variables.

Location	Means	Pair-Wise Comparison
arm	0.6715	arm vs calf: p<0.0001
calf	0.7313	calf vs thigh: p = 0.3116
thigh	0.711	arm vs thigh: p = 0.0155

## Discussion

This study highlights the interconnectedness of the fascial bands. These bands not only intertwined and connected with other fascial bands at different depths, but they also merged with the epimysium immediately beneath the subcutaneous layer and ascended superficially to intersect with the skin's dermis. As confirmed by multiple 2D images and 3D reconstructed renditions, the SQ fascial bands formed a coherent network of fibers that ensured various layers of the skin structurally and mechanically linked. These finding corroborates what others have reported in the literature. In a cadaveric study of the extremities, trunk, anterior chest wall, and neck, Abu-Hijleh MF [3] observed interconnecting and merging fascial membranes within the subcutaneous layer at all locations studied. Johnson D [7] and Nash [8] also observed fascial bands radiating toward the skin from deeper subcutaneous/epimysial layers to form adherent junctions with the dermis. These junctions termed “skin ligaments” were identified in the abdomen and the extremities far more frequently than what is commonly perceived as limited to certain locations (i.e. Zygomatic ligament in the cheek and Coopers ligament in the breast) [7]. This ubiquitous fascial network likely serves to maintain structural cohesiveness between the skin and the underlying muscle and bones. Without the tensile resilience afforded by the collagen fibers and without the structural junctions to serve as anchors, skin would otherwise be prone to disengaging and completely detaching from the body. Importantly, this structural arrangement also ensures that the skin and its structural components are not mechanically isolated. Substantial muscular movement or large skin displacements would generate mechanical strain along the fascial bands that can theoretically span well beyond a focal location and thus affect fibroblast mechanotransduction and even nerve activity at a large spatial scale. Within this framework, the fascial network may conceivably form a body-wide network that not only helps mediate mechanical forces but also cellular and nervous activities as well [9].

Subcutaneous blood vessels were also extensively integrated in this fascial network. In our ultrasound images at all three locations, SQ fascial bands were found to compartmentalize and encase the blood vessels (Figure 4). At the spatial resolution of our ultrasound, the vessel wall appeared inseparable from the fascial bands, particularly at the most superficial and deep aspect of the vessel. Past authors have equated this structural arrangement with the “Egyptian eye” and reported such morphologies at both the saphenous vein [10] and the cephalic vein [11]. For both these veins, the blood vessels were bounded by subcutaneous fascial bands superficially and by perimuscular fascia at a deeper level. Our images have identified similar arrangements, although subcutaneous vessels were at times bound by subcutaneous fascial bands on the deeper aspect of the vessel as well. From a clinical perspective, these fascial bands help prevent excessive dilatation of the veins and account for why veins constrained by fascia are less prone to develop varicosities relative to tributary veins without fascia sheathing [12]. Given the fact that fascial bands are contiguous with both skin and muscles, they may transmit mechanical forces arising from muscle contraction or from skin shear movement to the vessel wall and thus help modify blood flow in a manner that is beneficial to the affected limb. Moreover, the variable structural arrangements between the vein and fascial bands seen in Figure 4D may be strategically formed in such a way that only specific segments of the vein are affected by mechanical stresses.

Quantitative analyses of the ultrasound images revealed that the total SQ fascial band area was greatest at the calf and thigh and smallest at the arm. This difference may be attributed to the greater mechanical forces typically generated at the lower extremities. Daily ambulation and the need to chronically sustain the weight of the body are two conditions that likely facilitate a sustained increase in fascial band content. Future studies may consider evaluating how daily activities or weight training would affect the total fascial content in the subcutaneous layer, and whether greater total fascial band area would be identified in more active and possibly heavier individuals.

According to our quantitative analysis, the total fascial band area in the calf was statistically similar to that of the thigh, despite the thigh's thicker SQ zones (greater SQ fat layer). This decreased fascial band densities at the thigh was manifested by a comparatively greater number of fascial bands but an overall reduced fascial band thickness and was revealed by the fragmented and curved appearance of echogenic bands in Figure 3 panel T3 in contrast to fewer yet thicker bands seen in the calf images (Figure 3, Panel C1–C3). This inverse relationship between the average SQ fascial band thickness and SQ zone thickness was observed across body sites (including the arm) and across individuals as well (p<0.0001, Figure 6C). Interestingly, a statistically significant correlation between band number and SQ zone thickness was not observed when comparisons were made across individuals (p = 0.2543, Figure 6B). Why these patterns exist remains unclear, but one may speculate that thinner SQ layers would sustain greater amounts of mechanical (both shear and lateral) forces per volume and thus require thicker and more cohesive fascial bands. A thicker SQ layer, on the other hand, should dissipate such forces over its full depth and thus may not require the thicker, cohesive collagenous bands. The fact that band thickness was significantly correlated with SQ zone thickness and not total band number suggests that the SQ tissue preferentially alters the band's *thickness* rather than to change the *number* of bands to respond to these hypothetical mechanical forces. This, however, cannot be conclusively established without more microscopic imaging techniques, larger sample size, or more prospective data (e.g. temporal response to changes in mechanical force).

Paradoxically, in our study, increased average fascial band number was associated with an increase in fascial band thickness (Figure 6A). A reasonable functional explanation for this finding cannot be given, although it may reflect regional differences. The thigh, in particular, appears to have increased band thickness as the band number is increased while the other two regions did not.

To investigate the possible functional significance of the SQ fascial bands, we investigated the anisotropy of the SQ zone using a spatial coherence measure. In material science, anisotropy typically indicates a directional preference and implies the ability of a material to handle mechanical forces along a specific axis. In biological tissue, anisotropy exists either to facilitate a functional role or to adapt to persistent mechanical forces. Muscles, for instance, are characterized by high anisotropy ensuring that it is optimized for generating tensile force along a specific axis; bones are usually able to withstand greater tensile forces along the longitudinal axis compared to the transverse direction. Collagenous fibers, similarly, possess anisotropy and respond to mechanical force by aligning along the primary direction of tension. In a recent in vitro experiment involving collagen and fibroblasts, strong collagen fiber alignment and densification occurred in response to applied strains greater than 5% [13]. Fiber alignment was permanently imprinted when the material was cyclically stretched up to strains of 15%. Our in vivo analysis of SQ zone coherence suggests that, SQ anisotropy is significantly associated with SQ zone thickness and body site. Reduced SQ zone thickness was associated with increased SQ anisotropy and implies that mechanical stress is distributed across the subcutaneous tissue. Thinner subcutaneous tissues are more likely to concentrate the mechanical forces and thus are associated with increased spatial anisotropy. This multivariable analysis also reveals that the calf is associated with greater anisotropies and thus theoretically more likely to experience mechanical stress than the arm or thigh.

This study has a number of limitations. First, it involved a limited number of patients, evaluated three specific body locations, and did not have sufficient power to evaluate the effects of gender, age or ethnicity. Future studies may consider evaluating other body segments and other locations within the extremity of a larger, more diverse study cohort. Second, the study relied on static ultrasound images for characterizing the SQ fascial system. Functional, dynamic measures were not obtained. Third, spatial anisotropy is only a surrogate marker of mechanical force and cannot be interpreted as a direct measure of stress. Finally, the ultrasound device itself has technical limitations. Ultrasonography relies on heterogenic impedance of acoustic waves traveling axially from skin to deeper layers. Vertical structures poorly reflect acoustic waves traveling in an axial (vertical) trajectory and thus are not well characterized on the ultrasound images. This will generate an intrinsic anisotropy within the image that should be considered prior to interpreting the data.

Despite these limitations, this study has a number of advantages that adds to the existing, albeit limited, SQ fascia literature. In vivo ultrasonography was used to quantify and characterize the SQ fascial bands – a method that is more likely to be valid to real-life physiology than cadaveric-dissection studies. In addition, novel techniques incorporating principal component analysis, three dimensional reconstruction, and spatial coherence measures were incorporated in this study to better reveal the morphological features of the SQ fascial bands. These methods and techniques collectively have revealed that SQ Fascia is an interconnected network that likely transmits mechanical forces over large spatial scales and across various tissues. The exact functional significance of this behavior remains unclear and awaits additional study.

## Supporting Information

Video S1 An animation of a three-dimensional cubic tissue with 360 degree rotation in the ImageJ software (National Institute of Health, USA); transparency was set at 70. The video shows a vein tunnel enclosed between a SQ fascial sheet and the epimysial sheet.
(AVI)
